# (*Z*)-2-Sulfanyl­idene-5-(thio­phen-2-yl­methyl­idene)imidazolidin-4-one

**DOI:** 10.1107/S1600536811033034

**Published:** 2011-08-27

**Authors:** Abdullah M. Asiri, Hassan M. Faidallah, Abdulrahman O. Al-Youbi, Tarik R. Sobahi, Seik Weng Ng

**Affiliations:** a Chemistry Department, Faculty of Science, King Abdulaziz University, PO Box 80203 Jeddah, Saudi Arabia; b Center of Excellence for Advanced Materials Research, King Abdulaziz University, PO Box 80203 Jeddah, Saudi Arabia; c Department of Chemistry, University of Malaya, 50603 Kuala Lumpur, Malaysia

## Abstract

The mol­ecule of the title compound, C_8_H_6_N_2_OS_2_, has a V shape with two five-membered rings attached to a methyl­ene C atom. All non-H atoms are approximately coplanar (r.m.s. deviation = 0.096 Å). In the crystal, mol­ecules are linked by N—H⋯O hydrogen bonds into layers. The thio­phene ring is disordered over two positions; the major orientation has an occupancy of 0.683 (3). is there an intramolecular N---H...S bond?

## Related literature

For two 5-aryl-2-thioxoimidazolin-4-ones, see: Chowdhry *et al.* (2000 ▶); Książek *et al.* (2009 ▶).
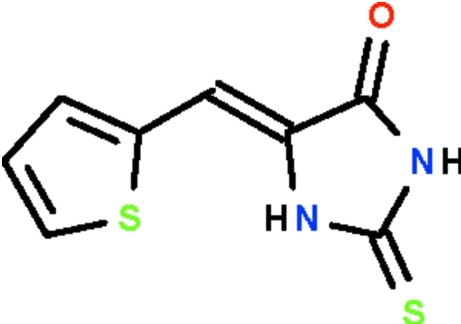


## Experimental

### 

#### Crystal data

C_8_H_6_N_2_OS_2_
*M* *_r_* = 210.27
Triclinic, 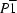

*a* = 6.1022 (6) Å
*b* = 7.0806 (8) Å
*c* = 11.0425 (13) Å
α = 72.582 (11)°
β = 76.116 (10)°
γ = 75.640 (9)°
*V* = 433.87 (8) Å^3^
*Z* = 2
Cu *K*α radiation
μ = 5.22 mm^−1^
*T* = 100 K
0.25 × 0.20 × 0.02 mm

#### Data collection

Agilent SuperNova Dual diffractometer with an Atlas detector
Absorption correction: multi-scan (*CrysAlis PRO*; Agilent, 2010 ▶) *T*_min_ = 0.356, *T*_max_ = 0.903
2599 measured reflections
1677 independent reflections
1519 reflections with *I* > 2σ(*I*)
*R*_int_ = 0.027

#### Refinement

*R*[*F* ^2^ > 2σ(*F* ^2^)] = 0.043
*wR*(*F* ^2^) = 0.122
*S* = 1.04
1677 reflections
134 parameters
6 restraints
H-atom parameters constrained
Δρ_max_ = 0.33 e Å^−3^
Δρ_min_ = −0.44 e Å^−3^

### 

Data collection: *CrysAlis PRO* (Agilent, 2010 ▶); cell refinement: *CrysAlis PRO*; data reduction: *CrysAlis PRO*; program(s) used to solve structure: *SHELXS97* (Sheldrick, 2008 ▶); program(s) used to refine structure: *SHELXL97* (Sheldrick, 2008 ▶); molecular graphics: *X-SEED* (Barbour, 2001 ▶); software used to prepare material for publication: *publCIF* (Westrip, 2010 ▶).

## Supplementary Material

Crystal structure: contains datablock(s) global, I. DOI: 10.1107/S1600536811033034/bt5612sup1.cif

Structure factors: contains datablock(s) I. DOI: 10.1107/S1600536811033034/bt5612Isup2.hkl

Supplementary material file. DOI: 10.1107/S1600536811033034/bt5612Isup3.cml

Additional supplementary materials:  crystallographic information; 3D view; checkCIF report

## Figures and Tables

**Table 1:** Hydrogen-bond geometry (Å, °)

*D*—H⋯*A*	*D*—H	H⋯*A*	*D*⋯*A*	*D*—H⋯*A*
N2—H2⋯O1^i^	0.88	2.20	2.873 (2)	133

Symmetry code: (i) 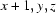.

**Fig. 1.**
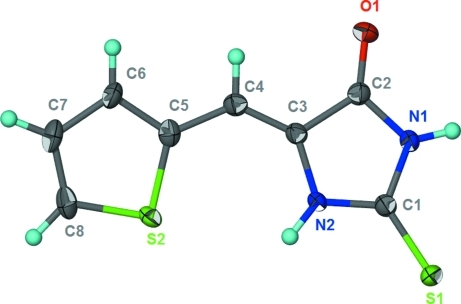
Ansiotropic displacement ellipsoid plot (Barbour, 2001) of C8H6N2OS2 at the 70% probability level; hydrogen atoms are drawn as spheres of arbitrary radius. The disorder in the thienyl ring is not shown.

## supplementary crystallographic information

### Comment

The crystal structures of only a small number of 5-aryl-2-thioxoimidazolidin-4-ones have been reported, with that of the phenyl homolog been described only recently. The bond dimensions of the parent compound are used to explain the nature of the products of its cycloaddition reaction (Książek *et al.*, 2009). The 2-pyridyl derivative is also a flat molecule (Chowdhry *et al.*, 2000) The thienyl analog (Scheme I) is similarly planar (r.m.s. deviation 0.096 Å). The molecule has a somewhat butterfly shape with the two five-membered rings attached to the methylene carbon (Fig. 1). The –NH– unit at the 4-position (of one ring) points towards the S atom in the 2-position (of the other ring); however, the interaction is too weak to lock the molecule so that the thienyl ring is able to adopt two orientations. Two molecules are linked by an N–H···O hydrogen bond across a center-of-inversion to form a dimer.

### Experimental

Thiophene-2-carboxaldehyde (1.10 g, 10 mmol) in ethanol (20 ml) was added to a solution of the 2-thiohydantoin (1.16 g,10 mmol) in 20% ethanolic potassium hydroxide (20 ml). The mixture was stirred for 6 h. The mixture was then poured into water (200 ml). The precipitate that separated when this was acidified with 10% hydrochloric acid was collected and recrystallized from ethanol.

### Refinement

H-atoms were placed in calculated positions [C—H 0.95 0.98 and N—H 0.88 Å; *U*_iso_(H) 1.2*U*_eq_(C,N)] and were included in the refinement in the riding model approximation.

The thienyl ring is disordered over two positions; pairs of bond distances were restrained to within 0.01 Å of each other, and the displacement parameters of the overlaying atoms were set to be equal. The major disorder component refined to 68.3 (3)%.

The intensity measurements are complete to 95%; however, they are 100% complete at a 2θ limit of 135 °.

### Crystal data

**Table:**

C_8_H_6_N_2_OS_2_	*Z* = 2
*M**_r_* = 210.27	*F*(000) = 216
Triclinic, *P*1	*D*_x_ = 1.610 Mg m^−^^3^
Hall symbol: -P 1	Cu *K*α radiation, λ = 1.54184 Å
*a* = 6.1022 (6) Å	Cell parameters from 1386 reflections
*b* = 7.0806 (8) Å	θ = 4.3–73.9°
*c* = 11.0425 (13) Å	µ = 5.22 mm^−^^1^
α = 72.582 (11)°	*T* = 100 K
β = 76.116 (10)°	Plate, yellow
γ = 75.640 (9)°	0.25 × 0.20 × 0.02 mm
*V* = 433.87 (8) Å^3^	

### Data collection

**Table:**

Agilent SuperNova Dual diffractometer with an Atlas detector	1677 independent reflections
Radiation source: SuperNova (Cu) X-ray Source	1519 reflections with *I* > 2σ(*I*)
Mirror	*R*_int_ = 0.027
Detector resolution: 10.4041 pixels mm^-1^	θ_max_ = 74.1°, θ_min_ = 4.3°
ω scans	*h* = −4→7
Absorption correction: multi-scan (*CrysAlis PRO*; Agilent, 2010)	*k* = −8→8
*T*_min_ = 0.356, *T*_max_ = 0.903	*l* = −11→13
2599 measured reflections	

### Refinement

**Table:**

Refinement on *F*^2^	Primary atom site location: structure-invariant direct methods
Least-squares matrix: full	Secondary atom site location: difference Fourier map
*R*[*F*^2^ > 2σ(*F*^2^)] = 0.043	Hydrogen site location: inferred from neighbouring sites
*wR*(*F*^2^) = 0.122	H-atom parameters constrained
*S* = 1.04	*w* = 1/[σ^2^(*F*_o_^2^) + (0.0832*P*)^2^ + 0.1141*P*] where *P* = (*F*_o_^2^ + 2*F*_c_^2^)/3
1677 reflections	(Δ/σ)_max_ = 0.001
134 parameters	Δρ_max_ = 0.33 e Å^−^^3^
6 restraints	Δρ_min_ = −0.44 e Å^−^^3^

### Fractional atomic coordinates and isotropic or equivalent isotropic displacement parameters (Å^2^)

**Table:**

	*x*	*y*	*z*	*U*_iso_*/*U*_eq_	Occ. (<1)
S1	0.82425 (8)	0.32671 (8)	0.07136 (5)	0.0212 (2)	
S2	0.7731 (2)	0.1352 (3)	0.61560 (16)	0.0177 (3)	0.683 (3)
O1	0.0398 (3)	0.3931 (2)	0.34150 (16)	0.0214 (4)	
N1	0.3814 (3)	0.3582 (3)	0.19518 (18)	0.0165 (4)	
H1	0.3243	0.3831	0.1247	0.020*	
N2	0.6346 (3)	0.2730 (3)	0.32364 (17)	0.0155 (4)	
H2	0.7665	0.2340	0.3513	0.019*	
C1	0.6123 (4)	0.3176 (3)	0.1982 (2)	0.0161 (4)	
C2	0.2501 (4)	0.3552 (3)	0.3155 (2)	0.0165 (5)	
C3	0.4173 (3)	0.2981 (3)	0.4032 (2)	0.0153 (4)	
C4	0.3529 (4)	0.2791 (3)	0.5309 (2)	0.0172 (5)	
H4	0.1912	0.3066	0.5592	0.021*	
C5	0.484 (2)	0.225 (8)	0.6318 (12)	0.017 (2)	0.683 (3)
C6	0.388 (2)	0.234 (2)	0.7571 (9)	0.0201 (7)	0.683 (3)
H6	0.2288	0.2766	0.7856	0.024*	0.683 (3)
C7	0.5563 (7)	0.1717 (7)	0.8394 (5)	0.0218 (9)	0.683 (3)
H7	0.5228	0.1714	0.9280	0.026*	0.683 (3)
C8	0.7709 (9)	0.1127 (9)	0.7737 (4)	0.0227 (12)	0.683 (3)
H8	0.9045	0.0645	0.8122	0.027*	0.683 (3)
S2'	0.3819 (11)	0.2103 (10)	0.7847 (4)	0.0201 (7)	0.317
C5'	0.494 (4)	0.210 (17)	0.628 (2)	0.017 (2)	0.317
C6'	0.731 (3)	0.159 (3)	0.6117 (17)	0.0177 (3)	0.317
H6'	0.8277	0.1592	0.5304	0.021*	0.317 (3)
C7'	0.816 (3)	0.106 (2)	0.7307 (10)	0.0227 (12)	0.317
H7'	0.9731	0.0609	0.7379	0.027*	0.317 (3)
C8'	0.6415 (17)	0.1274 (17)	0.8312 (14)	0.0218 (9)	0.317
H8'	0.6628	0.0994	0.9177	0.026*	0.317 (3)

### Atomic displacement parameters (Å^2^)

**Table:**

	*U*^11^	*U*^22^	*U*^33^	*U*^12^	*U*^13^	*U*^23^
S1	0.0143 (3)	0.0310 (3)	0.0138 (3)	−0.0020 (2)	−0.0009 (2)	−0.0024 (2)
S2	0.0127 (7)	0.0198 (7)	0.0184 (5)	−0.0001 (5)	−0.0058 (5)	−0.0018 (4)
O1	0.0122 (7)	0.0315 (9)	0.0211 (9)	−0.0048 (6)	−0.0035 (6)	−0.0068 (7)
N1	0.0139 (8)	0.0223 (9)	0.0131 (9)	−0.0046 (7)	−0.0041 (7)	−0.0020 (7)
N2	0.0108 (8)	0.0213 (9)	0.0129 (9)	−0.0026 (6)	−0.0037 (7)	−0.0013 (7)
C1	0.0149 (10)	0.0173 (9)	0.0154 (11)	−0.0047 (7)	−0.0035 (8)	−0.0012 (8)
C2	0.0159 (10)	0.0186 (10)	0.0157 (11)	−0.0062 (8)	−0.0021 (8)	−0.0035 (8)
C3	0.0137 (10)	0.0162 (9)	0.0153 (11)	−0.0041 (7)	−0.0034 (8)	−0.0014 (8)
C4	0.0155 (10)	0.0182 (10)	0.0180 (11)	−0.0057 (8)	−0.0026 (8)	−0.0034 (8)
C5	0.0229 (15)	0.014 (7)	0.0159 (13)	−0.007 (2)	−0.0053 (11)	−0.0011 (14)
C6	0.0226 (7)	0.0262 (17)	0.011 (2)	−0.0063 (8)	−0.0009 (15)	−0.0042 (16)
C7	0.034 (3)	0.020 (2)	0.0125 (14)	−0.0076 (19)	−0.004 (2)	−0.0041 (14)
C8	0.031 (4)	0.0192 (13)	0.018 (3)	−0.0072 (17)	−0.012 (3)	0.003 (2)
S2'	0.0226 (7)	0.0262 (17)	0.011 (2)	−0.0063 (8)	−0.0009 (15)	−0.0042 (16)
C5'	0.0229 (15)	0.014 (7)	0.0159 (13)	−0.007 (2)	−0.0053 (11)	−0.0011 (14)
C6'	0.0127 (7)	0.0198 (7)	0.0184 (5)	−0.0001 (5)	−0.0058 (5)	−0.0018 (4)
C7'	0.031 (4)	0.0192 (13)	0.018 (3)	−0.0072 (17)	−0.012 (3)	0.003 (2)
C8'	0.034 (3)	0.020 (2)	0.0125 (14)	−0.0076 (19)	−0.004 (2)	−0.0041 (14)

### Geometric parameters (Å, °)

**Table:**

S1—C1	1.660 (2)	C5—C6	1.380 (13)
S2—C5	1.706 (9)	C6—C7	1.433 (13)
S2—C8	1.702 (5)	C6—H6	0.9500
O1—C2	1.224 (3)	C7—C8	1.368 (5)
N1—C1	1.373 (3)	C7—H7	0.9500
N1—C2	1.374 (3)	C8—H8	0.9500
N1—H1	0.8800	S2'—C8'	1.693 (9)
N2—C1	1.358 (3)	S2'—C5'	1.705 (17)
N2—C3	1.407 (3)	C5'—C6'	1.378 (16)
N2—H2	0.8800	C6'—C7'	1.438 (15)
C2—C3	1.476 (3)	C6'—H6'	0.9500
C3—C4	1.344 (3)	C7'—C8'	1.356 (9)
C4—C5'	1.430 (8)	C7'—H7'	0.9500
C4—C5	1.431 (5)	C8'—H8'	0.9500
C4—H4	0.9500		
			
C5—S2—C8	92.3 (3)	C4—C5—S2	125.2 (7)
C1—N1—C2	111.81 (19)	C5—C6—C7	112.6 (10)
C1—N1—H1	124.1	C5—C6—H6	123.7
C2—N1—H1	124.1	C7—C6—H6	123.7
C1—N2—C3	110.50 (17)	C8—C7—C6	111.1 (7)
C1—N2—H2	124.8	C8—C7—H7	124.4
C3—N2—H2	124.8	C6—C7—H7	124.4
N2—C1—N1	107.33 (18)	C7—C8—S2	112.7 (5)
N2—C1—S1	126.45 (16)	C7—C8—H8	123.6
N1—C1—S1	126.20 (17)	S2—C8—H8	123.6
O1—C2—N1	126.4 (2)	C8'—S2'—C5'	93.5 (8)
O1—C2—C3	128.6 (2)	C6'—C5'—C4	128.4 (16)
N1—C2—C3	104.99 (17)	C6'—C5'—S2'	109.6 (10)
C4—C3—N2	132.2 (2)	C4—C5'—S2'	121.5 (14)
C4—C3—C2	122.56 (19)	C5'—C6'—C7'	113.2 (14)
N2—C3—C2	105.19 (18)	C5'—C6'—H6'	123.4
C3—C4—C5'	128.4 (9)	C7'—C6'—H6'	123.4
C3—C4—C5	131.6 (5)	C8'—C7'—C6'	111.3 (16)
C3—C4—H4	114.2	C8'—C7'—H7'	124.4
C5'—C4—H4	117.3	C6'—C7'—H7'	124.4
C5—C4—H4	114.2	C7'—C8'—S2'	112.2 (13)
C6—C5—C4	123.6 (9)	C7'—C8'—H8'	123.9
C6—C5—S2	111.2 (6)	S2'—C8'—H8'	123.9
			
C3—N2—C1—N1	−4.2 (2)	O1—C2—C3—C4	0.3 (3)
C3—N2—C1—S1	174.52 (16)	N1—C2—C3—C4	−179.96 (19)
C2—N1—C1—N2	4.3 (2)	O1—C2—C3—N2	−179.7 (2)
C2—N1—C1—S1	−174.39 (16)	N1—C2—C3—N2	0.1 (2)
C1—N1—C2—O1	177.1 (2)	C6—C7—C8—S2	1.0 (8)
C1—N1—C2—C3	−2.7 (2)	C5—S2—C8—C7	0.0 (18)
C1—N2—C3—C4	−177.4 (2)	C6'—C7'—C8'—S2'	−0.1 (18)
C1—N2—C3—C2	2.5 (2)		

### Hydrogen-bond geometry (Å, °)

**Table:**

*D*—H···*A*	*D*—H	H···*A*	*D*···*A*	*D*—H···*A*
N2—H2···O1^i^	0.88	2.20	2.873 (2)	133

Symmetry codes: (i) *x*+1, *y*, *z*.

## References

[bb1] Agilent (2010). *CrysAlis PRO* Agilent Technologies, Yarnton, Oxfordshire, England.

[bb2] Barbour, L. J. (2001). *J. Supramol. Chem.* **1**, 189–191.

[bb3] Chowdhry, M. M., Mingos, D. M. P., White, A. J. P. & Williams, D. W. (2000). *J. Chem. Soc. Perkin Trans. 1*, pp. 3495–3504.

[bb4] Książek, W., Kieć-Kononowicz, K. & Karolak-Wojciechowska, J. (2009). *J. Mol. Struct.* **921**, 109–113.

[bb5] Sheldrick, G. M. (2008). *Acta Cryst.* A**64**, 112–122. 10.1107/S0108767307043930 18156677

[bb6] Westrip, S. P. (2010). *J. Appl. Cryst.* **43**, 920–925.

